# Sporotrichosis

**Published:** 1909-01-23

**Authors:** 


### DERMATOLOGY.

### SPOROTRICHOSIS.

Sporotrichosis is the name given to an affection characterised by the appearance of indolent gummatous-like lesions in the subcutaneous tissue the result of invasion by a parasite belonging to the group of fungi known as sporotrichium. Its recognition is of relatively recent date, but it seems probable that it is far from being an uncommon disease, cases having hitherto been diagnosed as syphilis or tuberculosis. The earliest cases were reported in America in 1898 and 1900, but during the past two years some thirty or forty examples have been recorded in France, where the disease—its clinical features, its histology, and its bacteriology—has been closely studied by many observers. No case has yet been brought to light in this country. The lesions of this malady occur commonly as multiple subcutaneous nodules, which ultimately break down and discharge a purulent fluid through a fistulous opening. Sometimes the fistulous opening enlarges, and a fungating or crusted ulcerating nodule results. Some of these cases suggest syphilitic gummata, others tuberculous lesions. Surgical interference is found to be of little use, for, after incision and scraping, the lesions return, the borders of the wound becoming infiltrated and swollen. In another type of case there is formed a row of chronic abscesses along the arm, connected by a chain of chronic lymphangitis, and apparently originating from an infected finger. Quite recently cases have been described with intra-muscular abscesses, others with abscesses beneath the periosteum of the tibia, and others with involvement of the buccal mucous membrane. In most of the recorded cases a sporotrichial fungus has been obtained by cultivation from the lesions. This fungus—which has been called *Sporotrichium Beurmanii*—is grown readily upon ordinary media, although the most suitable is a glucose-peptone-agar. It grows best at ordinary room temperature and fully exposed to the air—*i.e.* without capping the tube. Colonies appear on the fourth to sixth day as small white acuminate points with a white marginal fringe. These slowly increase in size and become convoluted (much like the culture of ordinary favus), and eventually dark brown in colour, or black. Examination of films shows long filaments with numerous ovoid spores in groups or bunches. The presence of the fungus is not readily demonstrated in film preparations from the pus. Experimental inoculation of animals has fully demonstrated the pathogenic nature of the fungus, and it has lately been discovered that spontaneous sporotrichosis may occur in animals, in the rat more especially. The pathological anatomy of the lesion suggests in some cases syphilis, in others tuberculosis. Giant cells are often present in abundance. The fungus has been demonstrated in the lesions, readily in the experimental sporotrichosis of animals, but not so easily in human sporotrichosis. The rat when inoculated artificially suffers from visceral sporotrichosis, and there have been cases of human sporotrichosis in which visceral lesions have been suspected.

Two suggestions have been made as to the origin of this affection; one is that it is derived from animals suffering from the same disease, the other that it is derived from contact with infected vegetables. In support of this latter view are the facts that many cases have occurred in greengrocers, and that the fungus may be readily grown on vegetable matter, such as lettuce leaves.

The treatment of the affection is simple. The lesions clear up rapidly under the administration of iodide of potassium.

